# PKR-Like Endoplasmic Reticulum Kinase Is Necessary for Lipogenic Activation during HCMV Infection

**DOI:** 10.1371/journal.ppat.1003266

**Published:** 2013-04-04

**Authors:** Yongjun Yu, Francis J. Pierciey, Tobi G. Maguire, James C. Alwine

**Affiliations:** Department of Cancer Biology, Abramson Family Cancer Research Institute, Perelman School of Medicine, University of Pennsylvania, Philadelphia, Pennsylvania, United States of America; University of North Carolina at Chapel Hill, United States of America

## Abstract

PKR-like endoplasmic reticulum (ER) kinase (PERK) is an ER-associated stress sensor protein which phosphorylates eukaryotic initiation factor 2α (eIF2α) to induce translation attenuation in response to ER stress. PERK is also a regulator of lipogenesis during adipocyte differentiation through activation of the cleavage of sterol regulatory element binding protein 1 (SREBP1), resulting in the upregulation of lipogenic enzymes. Our recent studies have shown that human cytomegalovirus (HCMV) infection in human fibroblasts (HF) induces adipocyte-like lipogenesis through the activation of SREBP1. Here, we report that PERK expression is highly increased in HCMV-infected cells and is necessary for HCMV growth. Depletion of PERK, using short hairpin RNA (shRNA), resulted in attenuation of HCMV growth, inhibition of lipid synthesis and reduction of lipogenic gene expression. Examination of the cleavage of SREBP proteins showed PERK depletion inhibited the cleavage of SREBP1, but not SREBP2, in HCMV-infected cells, suggesting different cleavage regulatory mechanisms for SREBP1 and 2. Further studies showed that the depletion of SREBP1, but not SREBP2, reduced lipid synthesis in HCMV infection, suggesting that activation of SREBP1 is sufficient to induce lipogenesis in HCMV infection. The reduction of lipid synthesis by PERK depletion can be partially restored by expressing a Flag-tagged nuclear form of SREBP1a. Our studies also suggest that the induction of PERK in HCMV-infected cells stimulates SREBP1 cleavage by reducing levels of Insig1 (Insulin inducible gene 1) protein; this occurs independent of the phosphorylation of eIF2α. Introduction of an exogenous Insig1-Myc into HCMV infected cells significantly reduced HCMV growth and lipid synthesis. Our data demonstrate that the induction of PERK during HCMV infection is necessary for full activation of lipogenesis; this effect appears to be mediated by limiting the levels of Insig1 thus freeing SREBP1-SCAP complexes for SREBP1 processing.

## Author Summary

HCMV, a β-herpesvirus, is a significant pathogen which infects most of the human population by puberty. Primary HCMV infection can be unnoticed in healthy people, but can be life threatening for the immunocompromised and it is the most important cause of congenital infection in the developed world, frequently leading to deafness, mental retardation and developmental disability. HCMV infection alters cellular signaling and metabolism in order to establish and maintain an optimal cellular environment that can accommodate the increased demands for nutrients, energy, and macromolecular synthesis that accompany viral infection. On the other hand, increased demands for nutrients, energy and increased protein loading to the ER can induce ER stress, particularly the unfolded protein response (UPR). HCMV induces the UPR in infected cells but also highly regulates its effects. Our recent studies showed HCMV infection can also induce adipocyte-like lipogenesis by activation of the transcription factor SREBP1. We now provide evidence that the induction of the UPR is connected to lipogenic activation during HCMV infection. We show that the ER stress sensor protein, PERK, is critical for lipogenic activation induced during HCMV infection.

## Introduction

Viruses rely on the host cells to make viral proteins, replicate viral genomes and produce infectious virions. All the building blocks and energy required for these biosynthetic activities are derived from intermediary metabolism in the host cells. It is important to understand how viral infection manipulates host cell metabolism since it may reveal new targets for antiviral therapy. Studies in the last few years have shown that infection of HCMV can cause dramatic alterations of glucose and glutamine metabolism in the host cells [1]–[3]. Induction of the adipocyte specific glucose transporter 4 (GLUT4), to replace the less efficient GLUT1, allows HCMV infected cells to significantly increase glucose uptake [4]. Coupled with increased glucose uptake, glycolysis is greatly upregulated [1]. However, instead of producing energy in the tricarboxylic acid (TCA) cycle, a large amount of the glucose-derived carbon exits the mitochondria in the form of citrate to be converted to cytosolic acetyl-CoA to support fatty acid synthesis, which is necessary to support the viral infection [2]. Our recent studies have shown that HCMV infection is able to induce adipocyte-like lipogenesis through the activation of SREBP1 [5].

**Figure 1:**
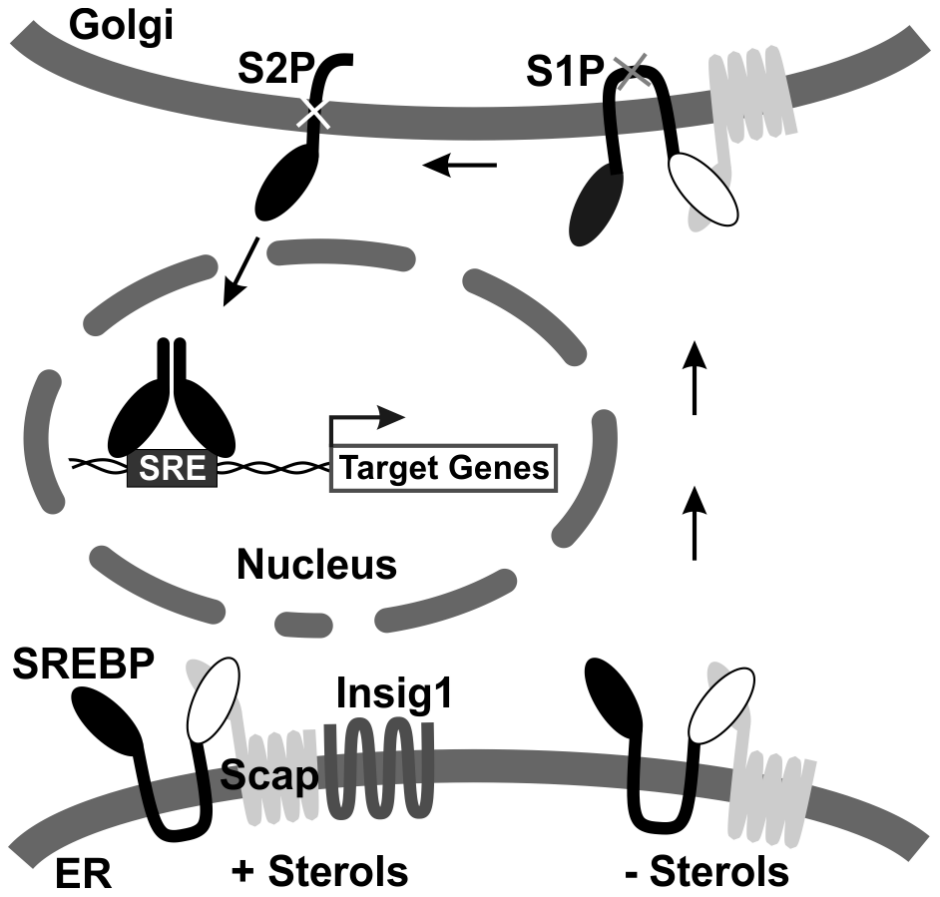
Diagram of SREBP maturation. See text for details. SREBP, sterol regulatory element binding protein; SCAP, SREBP cleavage activation protein; Insig1, insulin inducible gene 1.

SREBP proteins, the principle regulators of expression of genes involved in lipogenesis, belong to the basic helix-loop-helix leucine zipper (bHLH-Zip) family of transcriptional factors [6]. Unlike other members in this family, SREBPs are made as inactive precursors anchored in the membrane of the ER in complex with SCAP (SREBP cleavage activation protein) (Fig. 1). All SREBP precursors consist of a transcriptional domain at the N-terminus of approximately 500 amino acids containing the bHLH-Zip region, two hydrophobic transmembrane segments interrupted by a short segment (approximately 30 amino acids), and a regulatory domain at the C-terminus of approximately 590 amino acids [7]. In sterol-overloaded cells, SCAP binds to cholesterol which promotes binding to the ER membrane protein Insig1 (insulin inducible gene 1); under these conditions the SREBP-SCAP complex is retained in the ER by Insig1 and remains inactive (Fig. 1). In sterol-depleted cells, the interaction with Insig1 is weakened allowing COPII proteins to recruit the SREBP-SCAP complexes into COPII vehicles and export the complex to the Golgi apparatus where SREBPs are sequentially cleaved by S1P and S2P proteases to release the transcriptional domain (also called the mature SREBPs) that are translocated into the nucleus [8], [9] (Fig. 1). The mature SREBPs then bind to sterol regulatory elements in the promoters of lipogenic genes to activate their transcription and increase lipid synthesis [6]. Studies in our lab and others have shown that HCMV-infection can induce the cleavage of SREBP1 [5] and SREBP2 [10]. Our data also showed that the HCMV-induced cleavage of SREBPs, and the activation of the host cell lipogenic program, required the SCAP protein [5], thus the virus appears to be using the SCAP-mediated mechanism for SREBP transport and cleavage. However, the virus has altered this mechanism. In normal cells, the cleavage of SREBP1 is controlled by cellular sterol levels [6]; however, during HCMV infection the normal sterol feedback control is overridden and the cleavage of SREBP1 is constitutive regardless of high levels of sterols [5].

The ER responds to the accumulation of unfolded and/or misfolded proteins in its lumen by activating the UPR, which relieves the ER stress by reducing protein translation, degrading ER-localized mRNAs and misfolded proteins, as well as assisting protein folding. This is primarily accomplished through the activation of three ER-membrane associated sensor proteins: PKR-like ER kinase (PERK), inositol-requiring enzyme-1 (IRE1) and activating transcription factor-6 (ATF6) [11]. Under normal conditions, binding of the ER chaperone protein glucose regulated protein 78 (GRP78, also called BiP) to the ER luminal domains of PERK, IRE1 and ATF6 keeps these three proteins inactive. Following ER stress, BiP dissociates from the ER sensor proteins and preferentially binds to unfolded/misfolded proteins to assist in their folding. This leads to activation of PERK, IRE1 and ATF6 to induce the UPR. The activation of these three proteins affects broad aspects of cell fate and the metabolism of proteins, amino acids and lipids. However, it has been shown that HCMV modulates the effects of all branches of the UPR in order to maintain positive growth conditions for the virus [12], [13]. For example, our previous studies have shown that HCMV infection activates PERK, an eIF2α kinase, yet its role in eIF2α phosphorylation and translational attenuation is minimized in infected cells [12]. However, other functions of PERK have been reported, specifically, PERK is an important regulator of lipogenesis during adipocyte differentiation through activation of SREBP1 cleavage and upregulation of key lipogenic enzymes [14]. Thus we postulated that activation of PERK during HCMV infection leads to increased SREBP cleavage and lipogenic activation.

In the following study we show that PERK expression is significantly increased during HCMV infection and is essential for HCMV growth. Depletion of PERK in HCMV-infected cells resulted in inhibition of viral growth, lipid synthesis, expression of key lipogenic genes, and the cleavage of SREBP1.

## Results

### PERK expression is critical to HCMV growth

**Figure 2:**
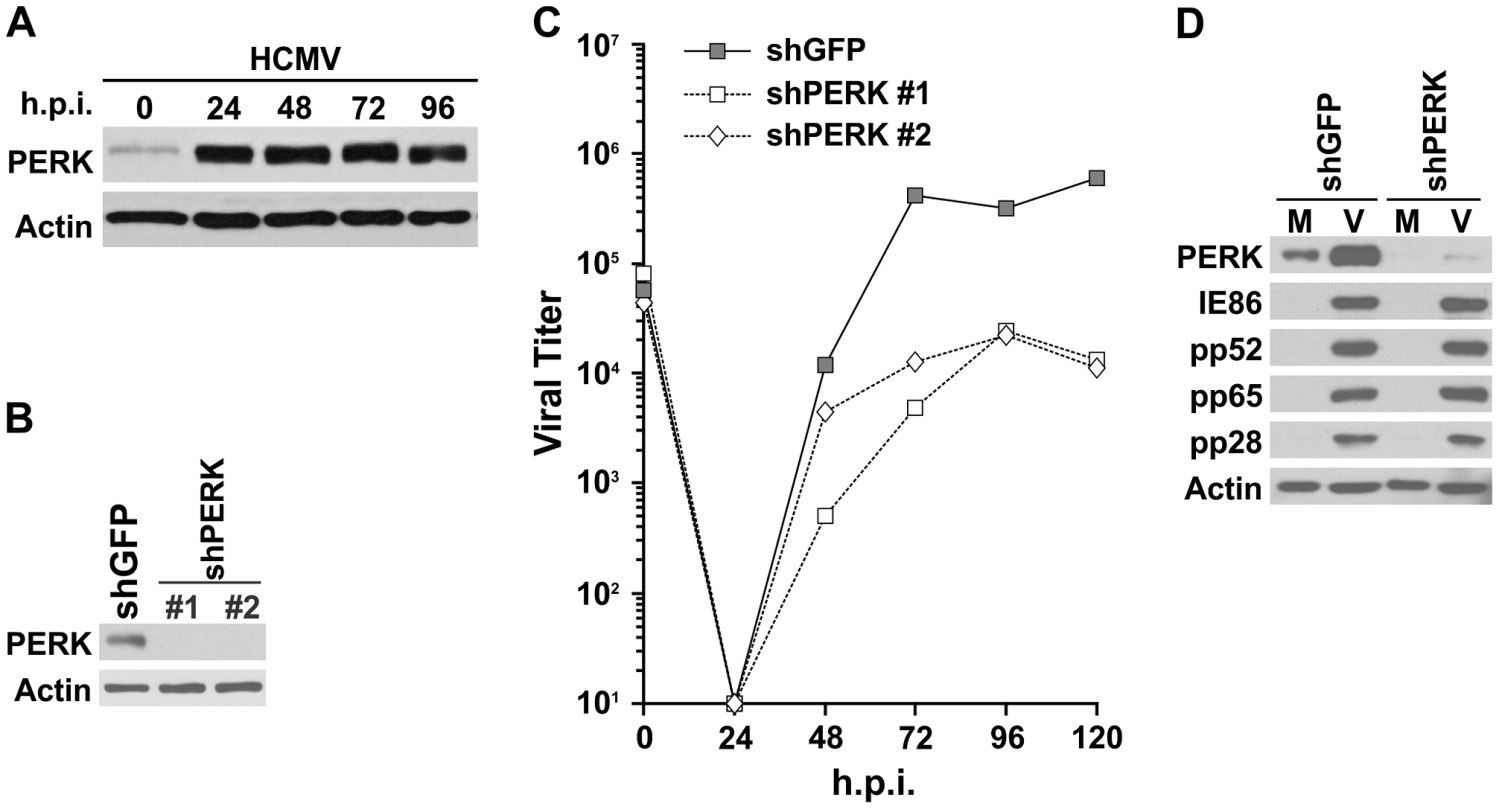
PERK expression is highly increased in HCMV-infected cells and critical for HCMV growth. (A) PERK protein levels in HCMV infection. Whole cell extracts from mock- or HCMV-infected cells at indicated times post infection were analyzed to determine the levels of PERK by Western. (B) Efficiency of shRNA treatment in depleting the levels of PERK protein in HF cells. Lentiviral vectors were used to introduce a control shRNA, shGFP, and two independent shRNAs targeted to PERK mRNA (shPERK, #1 and #2). Whole cell extracts were collected for Western analysis three days after shRNA treatment. (C) HCMV viral growth curves in the presence (shGFP) and absence of PERK (shPERK, #1 and #2) in HF cells. (D) HCMV viral protein expression in the presence (shGFP) and absence of PERK (shPERK #1) in HF cells.

Upon induction of the UPR, PERK activates itself by homodimerization and autophosphorylation [15], [16]. Our previous studies have shown that HCMV infection induces phosphorylation of PERK [12]. To determine whether PERK expression is elevated during HCMV infection, we examined PERK protein levels by Western analysis. HF cells were infected with HCMV and cell lysates were harvested at the indicated hours post infection (hpi). Figure 2A shows PERK protein levels were greatly increased at 24 hpi and remained at high levels throughout the infection time course to 96 hpi. In order to assess the importance of PERK induction during HCMV infection, we introduced a lentiviral vector expressing short hairpin RNAs (shRNA) against PERK mRNA (shPERK) to knockdown PERK expression, or a control shRNA against GFP mRNA (shGFP). Figure 2B shows that PERK expression can be eliminated efficiently by two independent shPERKs (#1 and #2) in HF cells after three days treatment. To test viral growth when PERK is depleted, HF cells were treated with shPERK for three days, followed by one day of serum starvation and then infection with HCMV at a multiplicity of infection (MOI) of 3. Viral samples were harvested at the indicated time points (Fig. 2C) and viral titers were determined using the 50% tissue culture infective dose (TCID_50_) method. In Figure 2C, the solid line indicates a normal HCMV growth curve in HF cells treated with the control shRNA, shGFP. The two dashed lines show the severe inhibition of HCMV growth upon depletion of PERK by shPERK #1 and #2 (Fig. 2C). The data indicate that PERK plays a critical role in HCMV lytic infection. We further tested viral protein levels in cells depleted of PERK. Whole-cell extracts were prepared from HCMV-infected cultures treated with shGFP or shPERK #1 at 72 hpi and evaluated by Western analysis. Figure 2D shows that the levels of an immediate-early protein (IE86), and early protein (pp52), as well as two late proteins (pp65 and pp28) were not altered in PERK-depleted cells, indicating that PERK depletion does not impact viral gene expression. These data show that the effects of PERK depletion on HCMV growth are not due to disruption of the temporal expression and accumulation of viral proteins, suggesting that the problems arise at the level of virion assembly, where lipid synthesis is required for membrane formation.

### PERK is required for HCMV-induced lipogenesis

**Figure 3:**
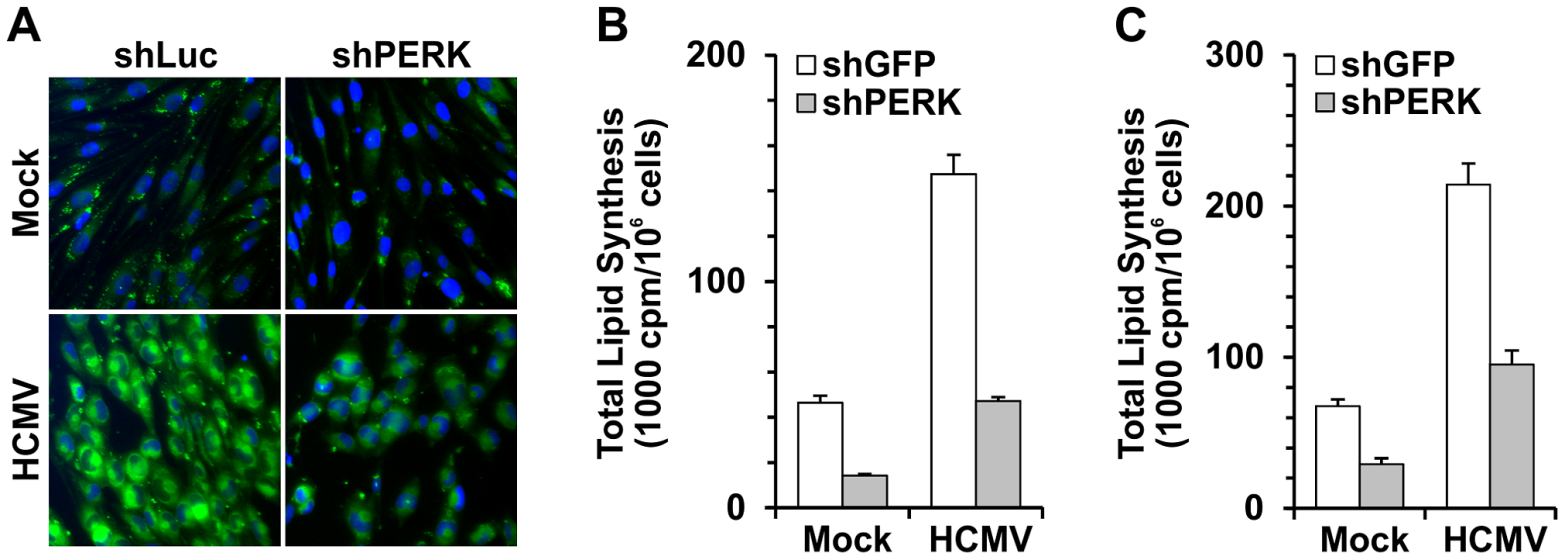
Depletion of PERK inhibits the induction of lipid synthesis in HCMV infection. (A) shLuc and shPERK treated HF cells, grown on fibronectin coated coverslips, were infected with HCMV and stained with lipophilic dye BODIPY 493/503 at 48 hpi. (B) and (C) Total lipid synthesis was assayed in HF cells. HF cells were treated with shGFP or shPERK, and then mock- or HCMV-infected for 48 hours, then the cells were labeled with ^14^C-acetate for 3 hours; total lipids were extracted and counted by scintillation counter.

We and others have reported that inhibition of lipid synthesis can inhibit HCMV growth [2], [5]. Beside its role in ER stress, PERK also serves as a critical regulator of lipid metabolism [14]. Given these data and the results in Figure 2, it is predicted that PERK might be essential for HCMV-induced lipogenesis. We examined cellular lipid levels using a fluorescent lipophilic dye BODIPY 493/503 to visualize lipid droplets [17]. Figure 3A shows that mock-infected HF cells expressing the control shRNA (shLuc) have low levels of lipid droplets and these are further reduced in PERK depleted cells. In agreement with our previous data [5], [18], the level of lipid droplets is greatly increased after HCMV infection; however, this is dramatically reduced in PERK-depleted cells (Fig. 3A). To further confirm the inhibition of lipid production by PERK depletion during HCMV infection, we also assayed total lipid synthesis by measuring incorporation of ^14^C-labeled acetate into lipids. Two independent assays showed that total lipid synthesis in HCMV-infected cells was about 3 times higher than that in mock-infected cells at 48 hpi (Fig. 3B and 3C). Consistent with lipid droplet staining, depletion of PERK resulted in a 50 to 65% reduction in total lipid synthesis in mock-infected cells. In HCMV-infected cells, total lipid synthesis was reduced 60–70% by PERK depletion (Fig. 3B and 3C).

**Figure 4:**
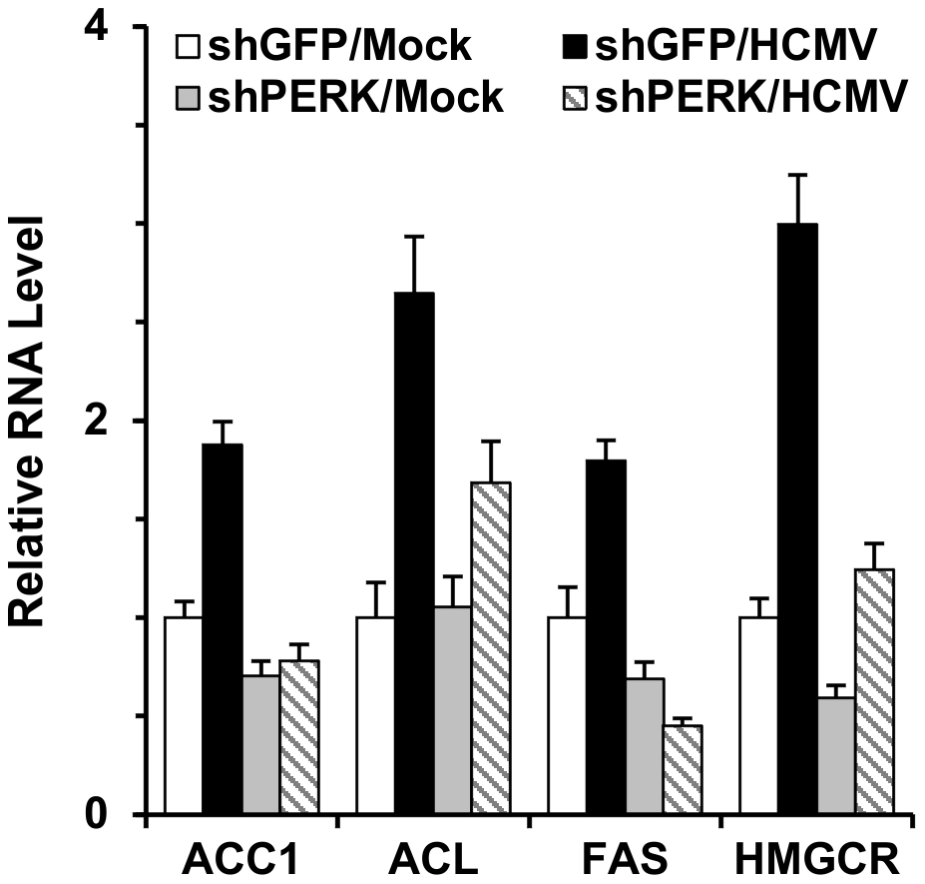
Depletion of PERK inhibits lipogenic gene expression in HCMV-infected HF cells. mRNA levels of lipogenic enzymes were determined by quantitative RT-PCR using total RNA extracted from mock- and HCMV-infected cells that had been treated with shGFP or shPERK at 72 hpi. ACC1, acetyl CoA carboxylase 1; ACL, ATP-citrate lyase; FAS, fatty acid synthetase; HMGCR, 3-hydroxy-3-methylglutaryl-CoA reductase.

Our previous data showed HCMV infection induced expression of key lipogenic genes [5]. We used quantitative RT-PCR to determine the levels of mRNAs encoding key lipogenic enzymes [acetyl-CoA carboxylase 1 (ACC1), ATP-citrate lyase (ACL), fatty acid synthetase (FAS) and HMG-CoA reductase (HMGCR)] in normal and PERK-depleted cells. Figure 4 shows that HCMV infection induces expression of ACC1, ACL, FAS, and HMGCR in cells treated with shGFP, confirming previous results [5]. However, the induction of these mRNAs by HCMV infection was substantially inhibited in PERK depleted cells (Fig. 4). The data in Figure 3 and 4 indicate that PERK is necessary for the full induction of lipogenesis in HCMV-infected cells.

### PERK depletion inhibits maturation of SREBP1, not SREBP2

**Figure 5:**
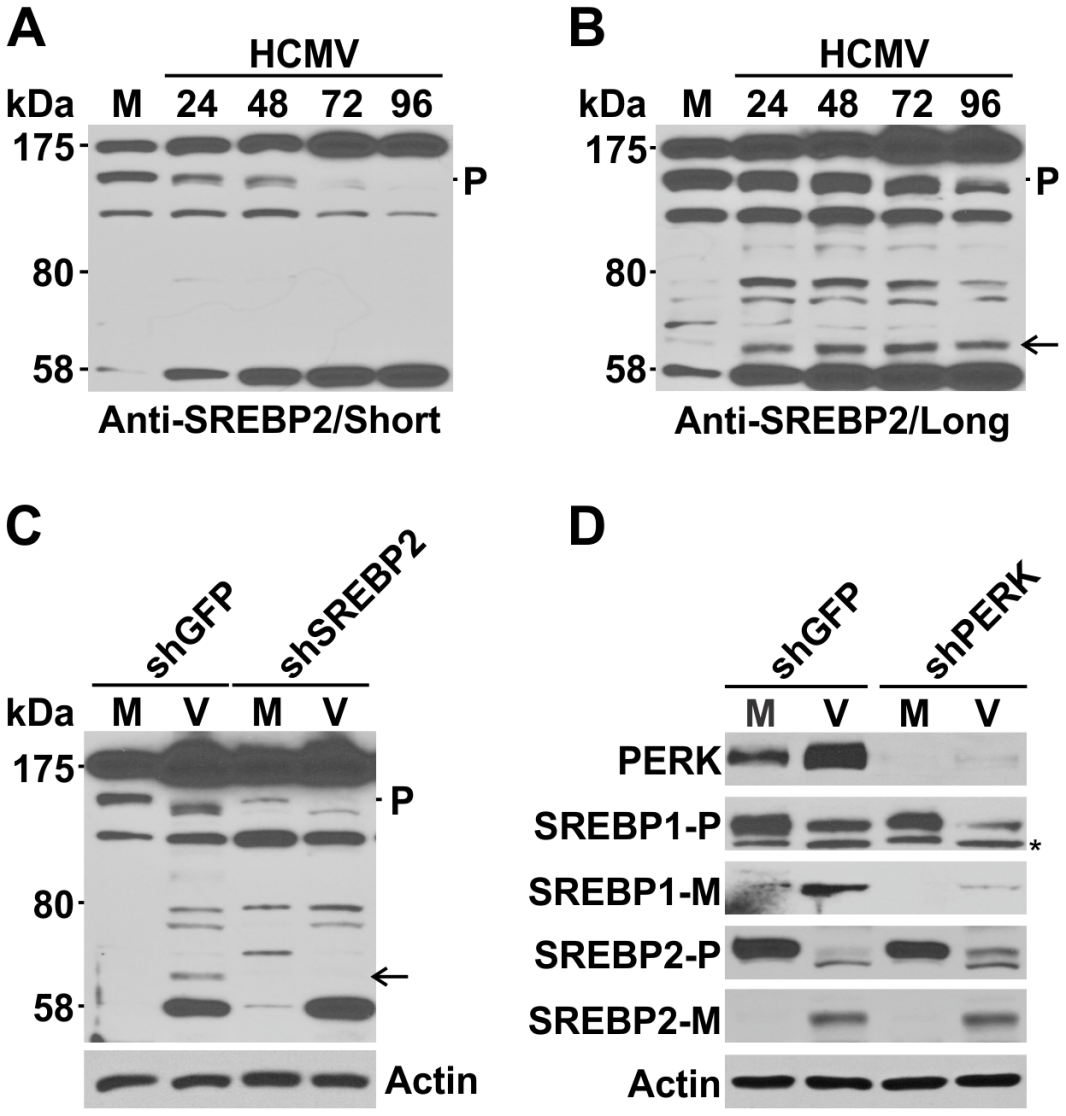
Depletion of PERK inhibits maturation of SREBP1. (A) and (B) Levels of SREBP2 in mock- and HCMV-infected cells. Whole cell extracts from mock- and HCMV-infected HF cells at the indicated times were analyzed to determine levels of SREBP2 precursor and mature form by Western. P, precursor SREBP2; Short, short exposure; Long, long exposure. (C) Whole cell extracts were prepared from HF cells treated with shGFP or shSREBP2 for three days and serum-starved for one day prior to mock- or HCMV-infection. Western analysis was performed by anti-SREBP2 antibody to determine the levels of the precursor and mature forms of SREBP2. M, mock infection; V, HCMV infection; P, precursor SREBP2; the arrow indicates the mature form of SREBP2. (D) The cleavage of SREBP1 and SREBP2 in PERK-depleted HF cells. HF cells were treated with shGFP or shPERK for 3 days, then mock- or HCMV-infected. Whole cell extracts were prepared at 48 hpi and analyzed by Western to determine the cleavage of SREBP1 and SREBP2. P, precursor; M, mature form; *. nonspecific protein.

The expression of lipogenic genes is regulated by the SREBPs. Mammalian cells have two genes encoding three SREBP isoforms: SREBP1a, SREBP1c and SREBP2 [19], [20]. SREBP1a and 1c are encoded by a single gene, *SREBF-1*, located on human chromosome 17p11.2 [21], [22] . The SREBP1a and 1c transcripts are produced through the use of alternative transcription start sites and only differ in their first exon resulting in a loss of 30 amino acids at the N-terminus of SREBP1c; SREBP1a and 1c cannot be differentiated immunologically. In cultured human fibroblasts, the SREBP1a transcript predominates [23].The three SREBP proteins control the expression of more than thirty genes involved in lipid metabolism [7]. SREBP2 predominantly upregulates genes involved in cholesterol biosynthesis and SREBP1c activates genes for fatty acid biosynthesis, while SREBP1a regulates gene expression for both fatty acid and cholesterol synthesis [24]. Previously, we have reported that SREBP1 is cleaved and activated in HCMV infection [5]. SREBP2 is also cleaved in HCMV infection, as reported by others [10]. Given the importance of PERK in lipid synthesis and induction of lipogenic genes during HCMV infection, we predicted that PERK depletion may inhibit activation of SREBPs. Cleavage of either SREBP1 or SREBP2 will produce mature forms of SREBPs with a size of approximately 60 kilodalton (kDa). Figure 5A shows a Western analysis of cell lysates from an HCMV infection time course probed with an anti-SREBP2 antibody. The antibody detects the 125 kDa SREBP2 precursor (P) as previously reported, the level of the precursor declines as the HCMV-infection proceeded [10]. Concurrently a protein band of approximately 58 kDa increases during HCMV-infection (Fig. 5A), and appears to be the mature form of SREBP2. However, a longer exposure of the same blot reveals an additional band migrating at about 60 kDa, slightly above the 58 kDa protein (Fig. 5B, arrow). To identify which band represents the real mature form of SREBP2, we examined the cleavage of SREBP2 in HCMV infected cells depleted of SREBP2 using an shRNA, shSREBP2. In mock- and HCMV-infected cells, shSREBP2 efficiently reduced the precursor (P) levels of SREBP2 compared to mock- or HCMV-infected cells treated with the shGFP control (Fig. 5C). Examination of the two proteins migrating at 58 and 60 kDa in infected cells showed that depletion of SREBP2 resulted in the loss of the less intense 60 kDa protein (arrow), while the 58 kDa protein band remained unchanged (Fig. 5C). Similar results were obtained in cells treated with two other shSREBP2s (Fig. S1A) which target different regions of the SREBP2 mRNA. Additionally, the 60 kDa but not the 58 kDa protein was not detected in cells depleted of SCAP protein (Fig. S1B) which is required for the cleavage of SREBP2 [6]. All these results show that the less intense 60 kDa band represents the mature form of SREBP2 and that the 58 kDa band represents a virally induced, cross reacting protein.

**Figure 6:**
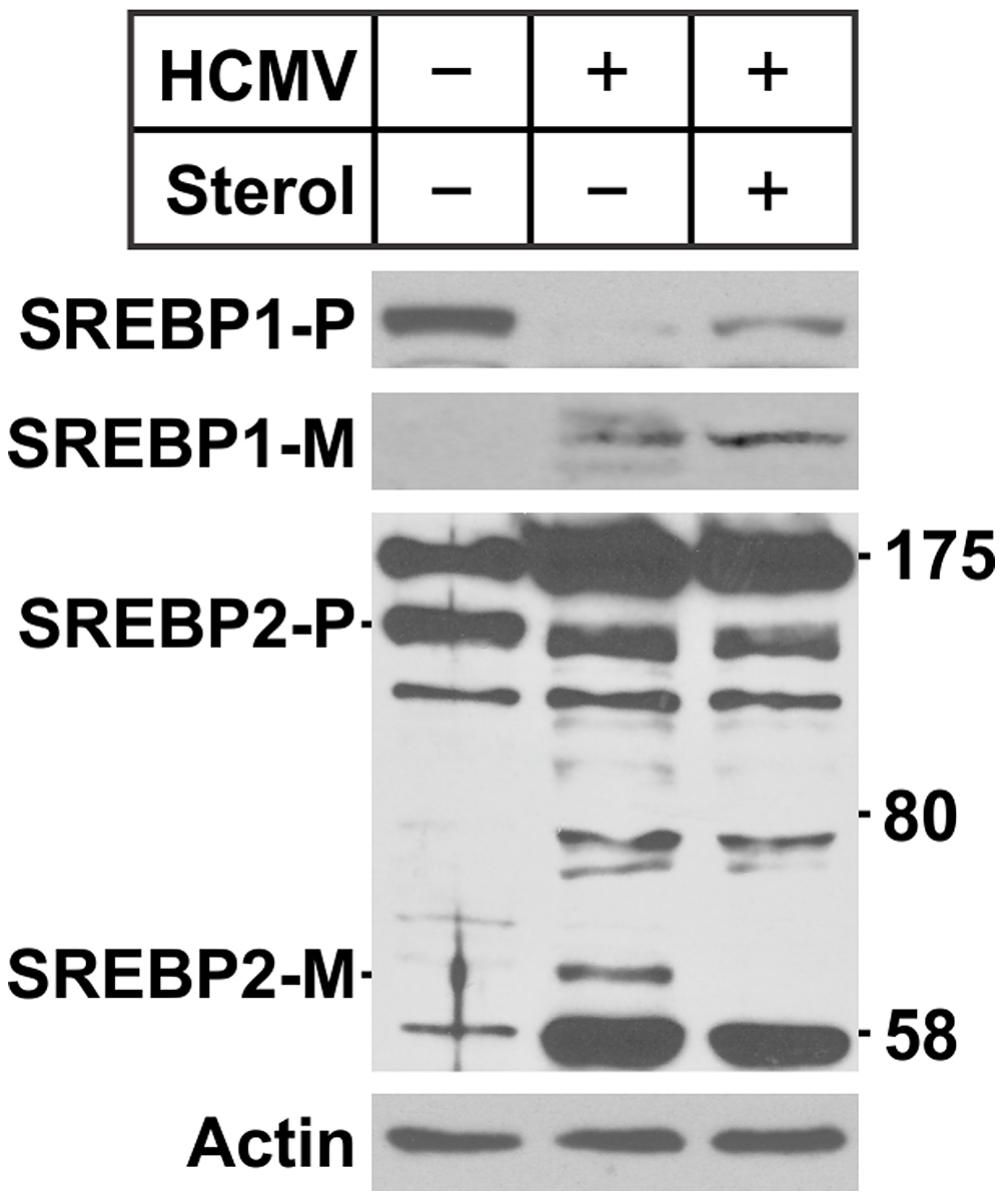
The cleavage of SREBP1 and 2 in the presence of sterol in HCMV-infected cells. Serum-starved HF cells were infected with HCMV or mock infected. At 2 hpi, the cells were either refed with serum-free medium alone or medium supplemented with 10 µg/ml cholesterol and 1 µg/ml 25-hydroxycholesterol. At 72 hpi, protein samples were harvested and tested for the effects of sterols on SREBP1 and 2 cleavage.

We next assessed the cleavage of both SREBP1 and SREBP2 in mock- and HCMV-infected cells treated with shGFP or shPERK. Normal precursor levels of SREBP1 and SREBP2 were detected in shGFP control treated cells; after HCMV infection, both SREBP1 and SREBP2 had decreased precursor levels but the mature forms accumulated as shown in Figure 5D. In mock-infected control cells treated with shGFP, a small amount of mature SREBP1 was detected and this was lost by depletion of PERK. In infected cells PERK depletion resulted in a severe reduction of the mature forms of SREBP1. Interestingly, neither the precursor nor the mature form of SREBP2 were affected by PERK depletion in mock- and HCMV-infected cells (Fig. 5D), indicating that SREBP1 and SREBP2 are cleaved by different mechanisms in HCMV infection, and PERK is necessary for the cleavage of SREBP1 only. The fact that SREBP1 and 2 are cleaved by different mechanisms during HCMV infection is supported by the data in Figure 6. We previously reported that SREBP1 can be cleaved successfully even in the presence of high level of sterols in HCMV infected cells, indicating that the normal sterol feedback control of the SREBP1 maturation is overridden by HCMV infection [5]. We examined the cleavage of SREBP2 in the same experiment. Figure 6 shows that SREBP1 is cleaved either in the presence or absence of supplemental sterols in HCMV-infected cells. However, under the same infection conditions the cleavage of SREBP2 was inhibited by the supplement of sterols. The difference in the maturation of SREBP1 and 2 may underlie their different roles in HCMV infection.

### Activation of SREBP1 is sufficient to induce lipogenesis by HCMV infection

**Figure 7:**
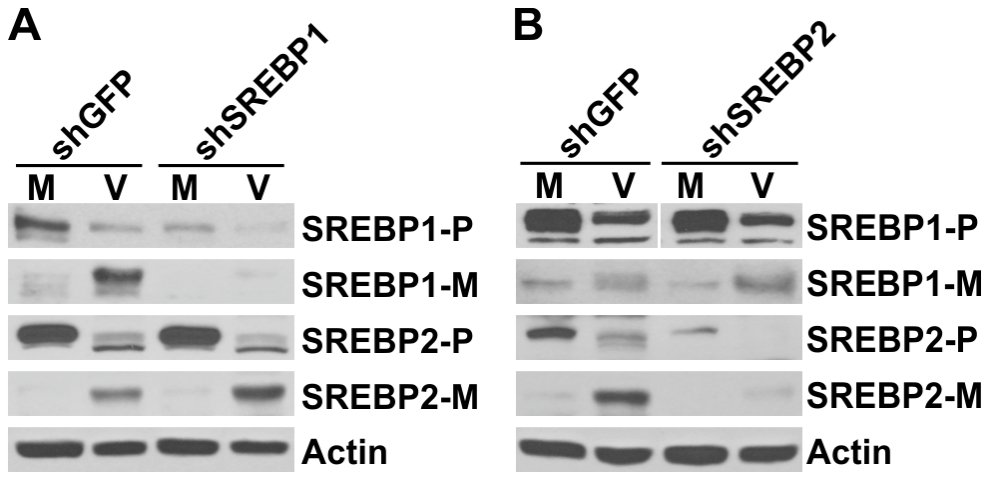
The cleavage of SREBP1 and SREBP2 in cells depleted of either SREBP1 or SREBP2, respectively. (A). HF cells were treated with shGFP or shSREBP1 for 3 days, then mock- or HCMV-infected. Whole cell extracts were prepared at 48 hpi and analyzed by Western to determine the levels of precursor and mature SREBP1 and SREBP2. (B) HF cells were treated with shGFP or shSREBP2 for 3 days, then mock- or HCMV-infected. Whole cell extracts were prepared at 48 hpi and analyzed by Western to determine the levels of precursor and mature SREBP1 and SREBP2. P, precursor; M, mature form.

Since SREBP1 and SREBP2 have different sets of target genes, they may play different roles in HCMV-induced lipogenesis. Thus we determined how lipid synthesis and HCMV growth were affected in cells that are depleted for either SREBP1 or SREBP2. We first determined how the depletion of one SREBP affected the levels of the other. Figure 7A shows that an shRNA against SREBP1 (shSREBP1) caused a dramatic loss of the precursors and mature forms of SREBP1. However, this loss of SREBP1 had no effect on the precursor level of SREBP2 in uninfected HF cells and there was a slight increase of the mature form of SREBP2 in HCMV-infected cells (Fig. 7A); this agrees with the results of SREBP1 knockout experiments [25]. A similar result was seen in SREBP2 depleted cells (Fig. 7B); depletion of SREBP2 reduced the precursor and mature form of SREBP2 in mock and infected cells, but had little effect on the levels of the SREBP1 precursors or mature forms. These data show that the depletion of one SREBP protein has no effect on the levels or cleavage of the other.

**Figure 8:**
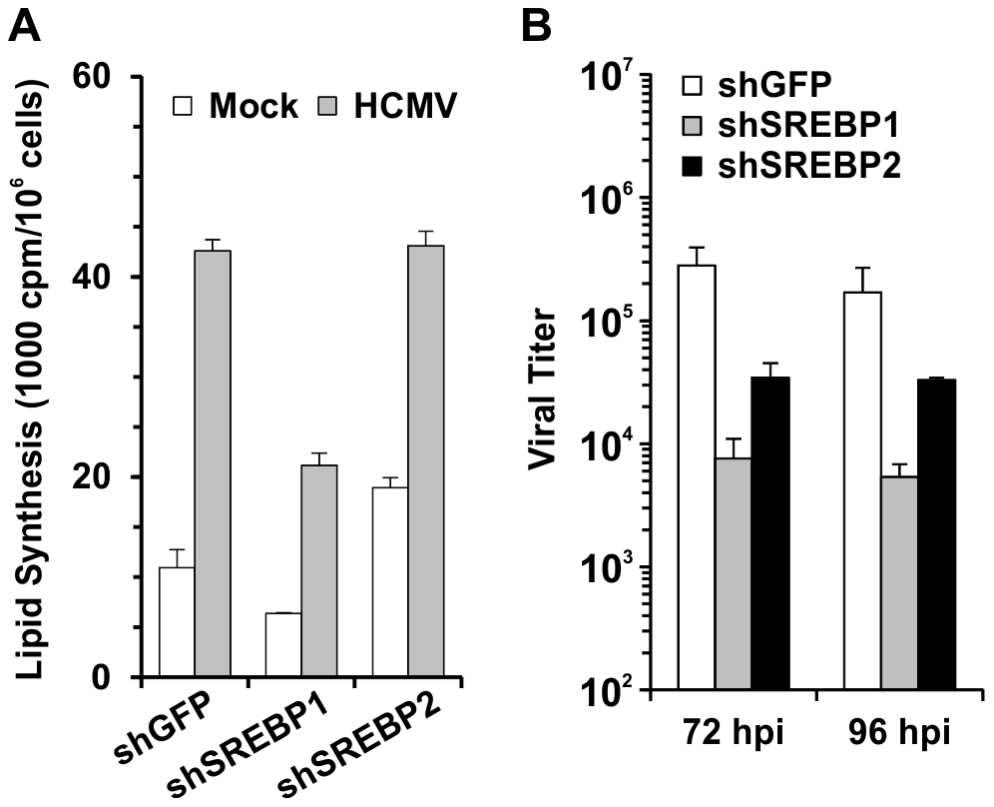
Activation of SREBP1 is sufficient to induce lipid synthesis. (A) Total lipid synthesis was measured in mock- and HCMV infected cells at 48 hpi that had been treated with shGFP, shSREBP1 or shSREBP2. Assays were performed as described in Figure 3B and 3C. (B) HCMV growth at 72 and 96 hpi was measured in HFs treated with shGFP, shSREBP1 or shSREBP2 as described in Materials and Methods.

We next tested total lipid synthesis in SREBP1 and SREBP2 individually depleted cells. Figure 8A showed that depletion of SREBP1 inhibited lipid synthesis in both mock and HCMV infected cells. Unexpectedly, the depletion of SREBP2 resulted in no reduction of lipid synthesis in HCMV-infected cells. In mock-infected cells, lipid synthesis was actually increased by the depletion of SREBP2, due to a compensatory effect of the loss of SREBP2 activity [26].

To further evaluate the importance of SREBP proteins in HCMV infection, we tested HCMV growth in SREBP1 or SREBP2 depleted cells. The viral growth data in Figure 8B show that either SREBP1 or SREBP2 depletion slowed HCMV growth in HF cells. However, SREBP1 depletion clearly had more severe effects than SREBP2 depletion. This further confirms that SREBP1 is playing a more central role in HCMV-induced lipogenesis than SREBP2.

### Expression of the SREBP1a nuclear form can partially restore lipid synthesis inhibited by PERK depletion

**Figure 9:**
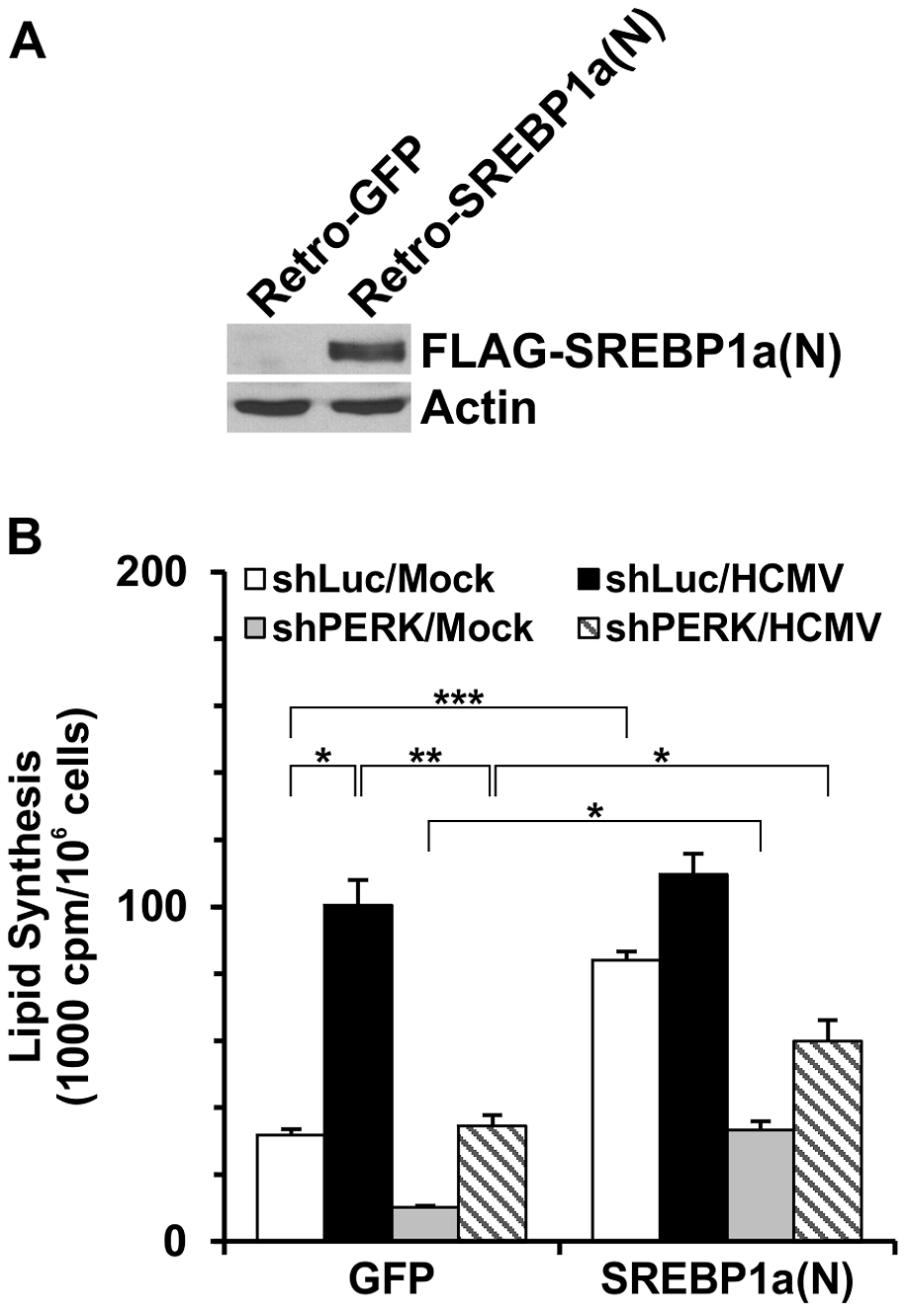
Restoration of HCMV induced lipid synthesis by expression of SREBP1a(N) in PERK-depleted HF cells. (A) Expression of Flag-tagged SREBP1a(N) in HF cells by retroviral transduction. Retro-GFP, retrovirus expressing GFP; Retro-SREBP1a(N), retrovirus expressing 2×FLAG-SREBP1a(N). (B) Total lipid synthesis in HF cells expressing SREBP1a(N). See text for details. * *P*<0.006; ** *P*<0.002; *** *P*<0.0005; *P* values were determined by Student's *t*-test.

As shown above, PERK depletion significantly slows HCMV growth and inhibits HCMV-induced lipogenesis; these correlate with reduction of the levels of the mature forms of SREBP1 in PERK-depleted, HCMV-infected cells. Thus we asked if lipid synthesis can be restored, at least partially, by expressing the nuclear (mature) form of SREBP1 in PERK-depleted, HCMV-infected cells. Since SREBP1a is the predominant transcript of the *SREBF-1* gene in fibroblasts [23], we used a retroviral vector to transduce a Flag-tagged SREBP1a nuclear form, 2×FLAG-SREBP1a(N), into HF cells. HF cells, 30–50% confluent, in 60 mm dishes were infected twice by retroviruses expressing 2×FLAG-SREBP1a(N) or GFP (control). After establishing these cells, they were tested for expression. Figure 9A shows good expression of 2×FLAG-SREBP1a(N) in HF cells after retroviral transduction.

The 2×FLAG-SREBP1a(N) and GFP expression cells were treated with lentiviral vectors expressing shLuc or shPERK for three days. The cells were then serum-starved for 24 hours and then infected with HCMV (MOI = 3). Lipid synthesis was assayed at 48 hpi. Figure 9B shows that in the control cells, transduced by the GFP expressing retrovirus and treated by shLuc, HCMV infection increased cellular lipid synthetic levels by greater than three fold compared to mock. PERK depletion greatly reduced the normal lipid synthetic rate in the mock infected cells and eliminated the activation by HCMV. These data agree with those shown in Figure 3B and 3C. In the cells transduced with the SREBP1a(N) expressing retrovirus and treated with shLuc, lipid synthesis was significantly increased compared to the GFP expressing controls. The lipid synthetic level was only modestly increased by HCMV infection in cells expressing SREBP1a(N) and shLuc; this is likely due to fact that the active SREBP1a is already in the nucleus of viral infected cells, maximally activating transcription of lipogenic genes. Interestingly, lipid synthesis was reduced about 60% by PERK depletion in mock-infected cells expressing SREBP1a(N). However, this level remains three times higher than that in mock-infected cells expressing GFP, demonstrating that SREBP1a(N) expression can, in part, compensate for the effects of PERK depletion. In PERK-depleted HCMV-infected cells, lipid synthesis was doubled by the expression of SREBP1a(N) compare to the expression of GFP, again showing that SREBP1a(N) expression can, in part, compensate for the effects of PERK depletion. However, the observation that SREBP1a(N) cannot fully compensate for PERK depletion, especially in mock-infected cells, suggests that PERK has additional effects on SREBP1 function or other aspects of lipid synthesis that remain to be discovered.

### PERK controls Insig1 levels

As shown in Figure 1, the SREBP1-SCAP complex is retained in the ER membrane by Insig1 when sterol levels are adequate. Thus an appropriate level of Insig1 must be maintained under these conditions to keep the SREBP1 in the uncleaved, inactive state. Importantly, Insig1 is a very unstable protein with a half-life shorter than 30 min [27]; thus the control of the Insig1 levels may be an important factor in the control of SREBP1 activation. In this regard, it is known that SREBP1 is cleaved more efficiently, and more of the active transcription factor accumulates in the nucleus, in Insig1 deficient cells [28]; and the cleavage of SREBP1 is also enhanced via direct depletion of Insig1 using siRNA [29].

**Figure 10:**
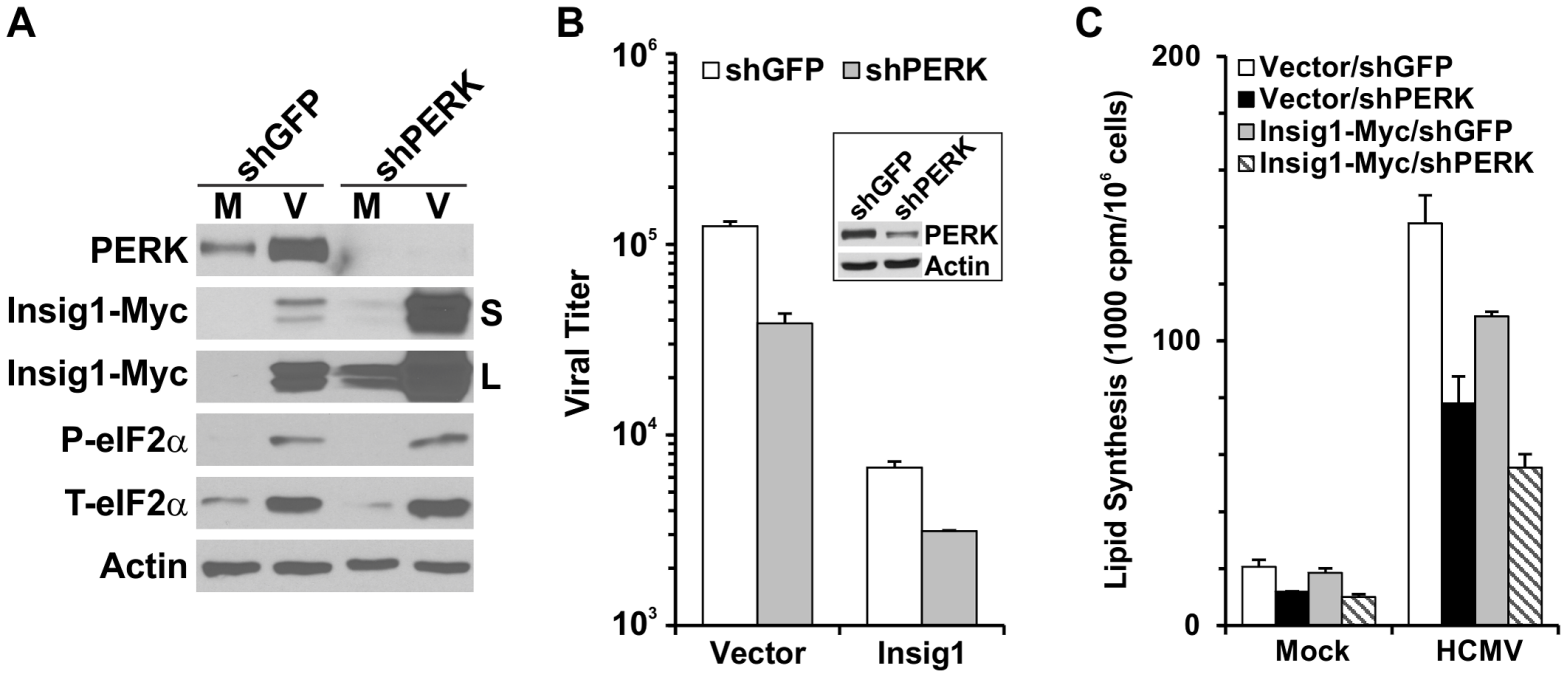
**Introduction** of Insig1-Myc inhibits HCMV growth and lipid synthesis in HCMV-infected cells. (A) The effect of PERK depletion on Insig1 levels and eIF2α phosphorylation. Human fibroblasts stably transfected with pCMV-Insig1-Myc were treated with shGFP or shPERK for three days, followed by one day serum starvation and then either mock- or HCMV-infected. Whole cell extracts were prepared for Western analysis at 48 hpi. Extracts were probed with anti-PERK, anti-Myc, anti-phospho-eIF2α, anti-total eIF2α and anti-actin. Two exposures of the Insig1-myc analysis are provided. S, short exposure; L, long exposure. (B) Introduction of exogenous Insig1-Myc inhibits HCMV growth. HF cells were treated with shGFP or shPERK for two days subsequent to transient transfection with pCMV-Insig1-Myc or the vector control (pCDNA3), then the cells were serum-starved for two hours and infected with HCMV. Viruses were harvested for titration at 72 hpi. See text for details. (C) Lipid synthesis in Insig1-Myc expressing HF cells. HF cells were treated as described in (B) prior to mock or HCMV infection. Total lipid synthesis was measured at 48 hpi.

Due to the lack of appropriate antibody to detect endogenous Insig1 in human cells, we introduced Myc-tagged Insig1 to examine if Insig1 protein levels could be altered due to the increase of PERK expression in HCMV infection. Plasmid pCMV-Insig1-Myc, a cDNA of Myc-tagged human Insig1 cloned in pCDNA3 vector [30], was electroporated into HF cells and stably transfected cells were selected using the drug G418. Pooled G418 resistant cells were treated with shGFP or shPERK for three days, followed by one day of serum starvation, then the cells were either mock- or HCMV-infected. Whole cell extracts were prepared for Western analysis at 48 hpi. In shGFP-treated, mock-infected cells the level of Insig1-Myc was nearly undetectable (a short, S, and a long, L, exposure of the Indig1-Myc Western are shown). In contrast, HCMV infection of these cells showed increased Insig1-Myc levels in the shGFP expressing cells, presumably due to HCMV-mediated transcriptional activation of the major immediate early promoter used in this expression vector (Fig. 10A). In shPERK-treated, mock-infected cells the depletion of PERK caused a significant increase in Insig1-Myc and this increase was even greater in the HCMV-infected cells. These results are consistent with a model where low levels of PERK result in increased Insig1 levels and high levels of PERK, as occurs in HCMV-infected cells, result in low levels of Insig1.

Recent data have shown that induction of the UPR by thapsigargin treatment induces a rapid loss of Insig1 in a PERK- and phospho-eIF2α-dependent manner [14]. This suggests that PERK's function as an eIF2α kinase may be involved. Thus we examined the phosphorylation status of eIF2α in the shGFP and shPERK treated cells. Figure 10A shows that levels of phospho (P)- and total (T)-eIF2α were increased during HCMV infection as we have previously reported [12]. However, there is no change in either total- or phospho-eIF2α levels comparing HCMV-infected cells expressing shGFP or shPERK. These data suggest that during HCMV infection phosphorylation of eIF2α is not mediated by PERK, in agreement with a recent report that the phosphorylation of eIF2α induced by HCMV infection is mediated, nearly exclusively, by the RNA activated protein kinase (PKR) [31]. Thus PERK's function to activate SREBP1 is independent of its eIF2α kinase activity during HCMV infection. The data in Figure 10A are consistent with the HCMV-mediated induction of PERK causing a reduction in endogenous Insig1 levels resulting in the increase of SREBP1 cleavage and lipid synthesis. Thus we predicted that increasing Insig1 levels through the introduction of an exogenous Insig1 would reduce HCMV growth and lipid synthesis in viral infected cells. To test this, we examined viral growth and total lipid synthesis in cells expressing exogenous Insig1-Myc. To maximize Insig1-Myc production we transiently transfected HF cells, via electroporation, with the plasmid expressing Insig1-Myc or its vector control, pCDNA3. Subsequent to electroporation the cells were infected with lentiviruses expressing shPERK or the control shGFP. In other experiments (Fig. 2D, 5D and 10A) the efficient depletion of PERK required three days of shPERK treatment and followed by one day of serum starvation prior to HCMV infection. Since the level of transiently transfected plasmid DNA decreases rapidly after transfection, we reduced the shPERK treatment. Hence, cells were cultured overnight at 37C after electroporation, and then treated with shPERK or shGFP for two days; this was followed by two hours of serum-starvation and then HCMV infection. Viruses were harvested at 72 hpi for titration and lipid synthesis was assayed at 48 hpi. The inset in Figure 10B shows that due to the shortened shPERK treatment the PERK protein level was reduced only about 60%, not as thoroughly as in other experiments. Nonetheless, this level of PERK depletion resulted in significant reduction in viral production in cells electroporated with the control vector plasmid (Fig. 10B). In shGFP treated control cells, the introduction of Insig1-Myc resulted in a 17-fold decrease in viral production. This is a substantial reduction considering that the Insig1-Myc was introduced by electroporation. Figure S2A shows a typical image of HF cells electroporated with a plasmid expressing red fluorescent protein (RFP) and three independent electroporations show that only 50 to 60% of the cells are transfected (Fig. S2B). The data suggest that increasing the levels of Insig1 in the infected cells can overcome the effects of induced PERK during infection; the further depletion of PERK in the Insig1-Myc transfected cells had little additional effect.

The total lipid synthesis assay results in Figure 10C show that in Mock-infected vector control cells, PERK depletion reduced lipid synthesis by approximately 40%, the introduction of Insig1-Myc into these cells only slightly reduced lipid synthesis and inhibition of lipid synthesis resulting from the combination of PERK depletion and by Insig1-Myc expression was additive. In vector-electroporated and HCMV-infected cells, lipid synthesis was reduced 40% by PERK depletion, recall that PERK was not totally depleted under the conditions used (Inset Fig. 10B). The introduction of Insig1-Myc into the shGFP treated, HCMV-infected cells significantly reduced total lipid synthesis, once more showing that increased production of Insig1 can reverse, at least partially, the effects of PERK induction in infected cells. Again it should be noted that the effect level of Insig1-Myc must be considered in the light that this is a transfections experiment where only a subset of the cells are overexpressing Insig1-Myc. The combination of Insig1-Myc expression and PERK depletion resulted in the greatest reduction of total lipid synthesis in infected cells.

All of the above data are consistent with a mechanism where the increased amounts of PERK in HCMV-infected cells results in lowering the endogenous levels of Insig1 which would promote the transport of the SREBP1-SCAP complex, cleavage of SREBP1 and the subsequent activation of lipogenic genes.

## Discussion

The activation of ER stress affects broad aspects of cell fate and metabolism, including lipid metabolism. Our data show that expression of PERK, an ER sensor protein, was highly elevated by HCMV infection and that this is critical for HCMV growth. Depletion of PERK resulted in decreased viral growth, lipid synthesis and expression of key lipogenic genes. Previous data have shown that induction of the UPR by thapsigargin treatment induces a rapid loss of Insig1 in a PERK- and phospho-eIF2α dependent manner [14]. Our data also suggest that a PERK mediated mechanism for controlling Insig1 levels is used by HCMV. Specifically, the increase in PERK production may result in the loss of endogenous Insig1 to further activate the cleavage of SREBP1 in HCMV-infected cells. In HCMV infected cells, this process is independent of PERK-mediated eIF2α phosphorylation since eIF2α is mainly phosphorylated by PKR in HCMV infection [31].

While our study shows that PERK depletion affects the SREBP1 cleavage pathway via Insig1, it also suggests that PERK may have other effects on SREBP expression and lipid synthesis. For example, we note that coincident with the reduction of the mature SREBP1 by PERK depletion, the level of the SREBP1 precursors is also decreased in HCMV-infected cells (Fig. 5D). We cannot rule out this result from PERK-mediated stabilization of the precursor and/or mature SREBP1. However, a transcriptional mechanism may be more likely since *SREBF-1* is a target gene of SREBP proteins, both nuclear forms of SREBP1 and SREBP2 can transcriptionally activate *SREBF-1* [7]. Thus the inhibition of SREBP1 maturation caused by PERK depletion would be expected to reduce *SREBF-1* transcription resulting in reduced levels of SREBP1 precursors. That PERK depletion affects *SREBF-1* transcription is supported by quantitative RT-PCR data (Fig. S3) which show RNA level of SREBP1 was reduced by PERK depletion in HCMV-infected cells. PERK is well known to have transcriptional effects via the transcription factor Nrf2 (NF-E2-related factor-2), a prosurvival transcription factor [32] which moves from the cytoplasm to the nucleus upon phosphorylated by PERK [32], [33]. However, previous studies from this lab have shown that Nrf2 is maintained in the cytoplasm during the course of HCMV infection despite PERK activation [34]. PERK also has an intrinsic lipid kinase activity, one function of which is to activate the multifunctional protein kinase Akt [35]. However, Akt is only temporally activated during the immediate-early/early phase of HCMV infection [36], [37] and the total protein levels of Akt decrease in late HCMV infection [37]. Thus it is questionable whether Akt affects lipid synthesis during infection; however, PERK's lipid kinase activity may have other functions related to the activation of lipid synthesis that are exploited by HCMV.

Previous studies have suggested that SREBP1 is more important for lipogenesis and adipocyte differentiation than SREBP2 [24]. Studies using transgenic and knockout mice showed that SREBP1a regulates gene expression related to both fatty acid and cholesterol synthesis, SREBP1c activates only genes related to fatty acid synthesis, and SREBP2 is more specific for genes related to cholesterogenesis [7]. Our data agree with these findings, in HCMV infected cells depletion of SREBP1 (both 1a and 1c) reduced total lipid synthesis while depletion of SREBP2 did not have the same effect. One explanation for this is that the transcriptional capacity of SREBP2 may be partially replaced by SREBP1a. Still, SREBP2 is not irrelevant during HCMV infection; however, clearly the virus grows better when SREBP1 is present.

In normal cells the three SREBP isoforms are matured via the same mechanism (Fig. 1) [24]. In HCMV-infected cells, however, the depletion of PERK reduced the level of the mature SREBP1 but had no effect on the level of the mature SREBP2. Thus SREBP1 and 2 are processed differentially in HCMV-infected cells. A difference in the processing mechanisms is also indicated by the observation that in infected cells excess sterols do not inhibit SREBP1 processing but do inhibit SREBP2 processing. Our data suggest that PERK induction in infected cells reduces the levels of Insig1, possibly the PERK effect is targeted to Insig1 in association with SREBP1-SCAP complexes and not SREBP2-SCAP complexes.

Our data suggest that neither the depletion of SREBP 1 nor SREBP2 can completely inhibit lipid synthesis; this may be due to incomplete depletion, partial overlap of functions or the involvement of other lipogenic transcription factors. In this regard, most lipogenic genes can also be activated by the carbohydrate responsive element-binding protein (ChREBP) [38]. ChREBP associates with Max-like protein X (Mlx) to serve as a glucose responsive transcriptional factor. It can work synergistically with SREBP1 to upregulate transcription of lipogenic genes. In mammalian cells, high level of nutrients, such as high glucose, can induce lipogenesis independent of SREBP1 [39], [40]. It has been recently reported that genetically engineered mice overexpressing GLUT4 can induce lipogenesis through upregulation of ChREBP in adipose tissues [41]. Our recent studies have shown that HCMV infection greatly increases GLUT4 expression [4], suggesting that ChREBP may be activated during HCMV infection, in addition to the SREBPs.

In conclusion, our data demonstrate that the induction of PERK during HCMV infection is necessary for full induction of lipogenesis. Depletion of PERK leads to the inhibition of the maturation of SREBP1, lipid synthesis and HCMV growth. Our data suggest that PERK mediates this effect by limiting the levels of Insig1, thus freeing SREBP1-SCAP complexes for SREBP1 processing. This function of PERK appears to be independent of its role as an eIF2α kinase.

## Materials and Methods

### Cell culture, viruses, reagents and plasmids

Primary and life-extended human foreskin fibroblasts (HFs) [42] were propagated and maintained in Dulbecco's modified Eagle's medium (DMEM) supplemented with 10% fetal calf serum, 100 U/ml penicillin, 100 µg/ml streptomycin, and 2 mM GlutaMAX (all reagents were obtained from Invitrogen). Cholesterol (C3045) and 25-hydroxycholesterol (H1015) were purchase from Sigma and used at concentrations described in Figure 6. The following plasmids were used in this study: pCMV-Insig1-Myc [30], pCDNA3.1-2×FLAG-SREBP1a(N) (Addgene plasmid 26801), pDsRed1-C1 (Clontech); pBabePuro-GFP was made by subcloning EGFP cDNA into BamHI and EcoRI sites of retroviral vector pBabe-Puro [43]. Lentiviral expression plasmids encoding for control shRNA shGFP [44], shLuc [4] were previously described. Lentiviral expression plasmids encoding for shPERK (TRCN0000001401, TRCN0000001399), shSREBP1 (TRCN0000020604) and shSREBP2 (TRCN0000020665, TRCN0000020667, TRCN0000020668) were purchased from OpenBiosystems. Plasmid electroporation into HF cells was performed using U-023 program for the Amaxa Nucleofector and electroporation kit for primary fibroblasts (Lonza) as described in the kit instruction.

HCMV (Towne strain) stocks were prepared and purified as previously described [36]. All HCMV experiments were performed in serum-starved HF cells by infection with a Towne strain of HCMV (MOI of 3) derived from a bacterial artificial chromosome clone modified to express green fluorescent protein (GFP) under the control of the simian virus 40 early promoter [45]. For lipid droplet staining using BODIPY 493/503, the wild type Towne strain of HCMV without the cassette containing the gene that encodes GFP was used. For viral growth assays, cells in 60 mm dishes were washed once with serum-free DMEM after 2 hours of viral incubation at 37C and then refed with 2 ml of serum-free DMEM. Viruses were harvested at indicated times and viral titers were determined using the TCID_50_ method. The experiments were set up in duplicate.

### Lipid droplet staining and lipid synthesis assay

Lipid droplets were stained with BODIPY 493/503 [4, 4-difluoro-1, 3, 5, 7, 8-pentamethyl-4-bora-3a, 4a-diaza-*s*-indacene] (Molecular Probes; Invitrogen) as described [17]. Briefly, confluent HF cells in 35 mm dishes were serum-starved for one day, then mock or HCMV infected in serum-free DMEM. At 48 hpi, cells were rinsed with ice-cold PBS and fixed with 4% paraformaldehyde for 30 min at room temperature, followed by three washes with PBS. Cover the cells with 1 ml of 1.0 µg/ml BODIPY 493/503 staining solution and incubate 10 min at room temperature, protected from ambient light. Wash the cells three times with PBS and mounted using Vectashield containing DAPI. The images were captured at the same microscopy exposure setting. Total lipid synthesis assay was performed as described previously [5].

### Retroviral vector

Retroviral vector pBabePuro-2×FLAG-SREBP1a(N) was made by subcloning cDNA of Flag-tagged SREBP1a nuclear form from pCDNA3.1-2×FLAG-SREBP1a(N) into BamHI and EcoRI sites in pBabe-Puro vector. Retroviruses, Retro-GFP and Retro-SREBP1a(N), were made as described [43]. Briefly, retroviral vector pBabePuro-GFP or pBabePuro-2×FLAG-SREBP1a(N) was cotransfected with amphotropic packaging plasmid QPSI (a gift from Dr. J. Alan Diehl, University of Pennsylvania) at a ratio of 1∶1 into 293T cells. Conditioned culture medium containing infectious retroviral particles was harvested at 24, 48 and 72 hours post-transfection, and used to infect exponentially growing HF cells in the presence of 8 µg/ml polybrene.

### RNA analysis

Total RNA was isolated using the RNeasy Mini Kit from Qiagene according to the manufacturer's protocol. Two µg of total RNA was used for first-strand cDNA synthesis by using SuperScript first-strand synthesis system (Invitrogen). For quantitative PCR, input cDNA was analyzed in triplate. All reactions were performed by using Taqman Universal PCR Master Mix kit in ABI 7900 PCR system (Applied Biosystems). The following primer sets (Applied Biosystems) were used: ACC1 (assay identification [ID] Hs01046047_m1), ACL (assay ID Hs00153764_m1), β-actin (assay ID Hs99999903-m1), FAS (assay ID Hs00188012_m1), HMGCR (assay ID Hs00168352_m1), and SREBP1 (assay ID Hs01088691_m1). Gene expression data were normalized to β-actin mRNA levels.

### shRNA experiments

Lentiviral vectors expressing shRNA were made as described [46]. Subconfluent HF cells were infected with lentiviral vectors in the presence of 8 µg/ml polybrene for 3–4 h, followed by the addition of fresh medium. For gene silence experiments, cells need to be treated with shRNA at least for 3 days and followed by one day of serum starvation, then infected with HCMV (MOI of 3) in serum-free DMEM for designed assays.

### Western analysis

Western blotting was performed by the procedures described previously [47]. The following antibodies were used in this study: anti-actin (MAB1501; Chemicon), anti-eIF2α (ab50733, Abcam), anti-ex2/3 [48], anti-Flag (MA1-91878, Thermo Scientific), anti-Myc (sc-789, Santa Cruz), anti-PERK (Ab65142, Abcam), anti-phospho-eIF2α (44-728G, Invitrogen), anti-pp28 (sc-56975, Santa Cruz), anti-pp52 (sc-69744, Santa Cruz), anti-pp65 (sc-52401, Santa Cruz), anti-SREBP1 (557036; BD Biosciences), anti-SREBP2 (Ab30682, Abcam).

### Accession numbers

GenBank accession and protein ID numbers of all genes/proteins mentioned in this study are listed in the following: ACC1 (NM_198834), ACL (NM_001096), Actin (NP_001091), eIF2α (NM_004094), FAS (NM_004104), HMGCR (NM_000859), IE86 (HCMV UL122, AAR31449.1), Insig1 (AY112745), PERK (NM_004836), pp28 (HCMV UL99, ACM48076.1), pp52 (HCMV UL44, ACM48032.1), pp65 (HCMV UL83, ACM48061.1), SREBP1a (NM_001005291), SREBP1c (NM_004176), and SREBP2 (NM_004599).

## Supporting Information

Figure S1 (A) Whole cell extracts were prepared at 48 hpi from HF cells treated with shGFP or three independent shRNAs targeting SREBP2 (TRCN0000020665, TRCN0000020667, TRCN0000020668) for three days and serum-starved for one day prior to mock- or HCMV-infection. Western analysis was performed by anti-SREBP2 antibody to determine the levels of the mature form of SREBP2. (B) The mature form of SREBP2 in SCAP-depleted cells. HF cells were treated with shGFP or shSCAP (TRCN0000078063) and infected with HCMV as described in (A), and then whole cell extracts were prepared. Western analysis was performed by anti-SREBP2 antibody to determine the levels of the mature form of SREBP2. M, mock infection; V, HCMV infection; the arrow indicates the mature form of SREBP2.
(TIF)

Figure S2 Transfection efficiency in HF cells via electroporation. (A) HF cells were transfected with a plasmid expressing RFP via electroporation. Two days after electroporation, cells on coverslips were fixed and stained with DAPI. The images were captured using a fluorescent microscope. (A) A representative image of electroporated HF cells. Red, RFP; blue, DAPI. (B) Average efficiency of three independent electroporations. After electropotation, fluorescent images were captured. Cells with RFP or DAPI signal were counted by Image-Pro 6.3 software and transfection efficiency was calculated.
(TIF)

Figure S3 SREBP1 mRNA levels in PERK-depleted HF cells. mRNA levels of SREBP1 were determined by quantitative RT-PCR using total RNA extracted from mock- and HCMV-infected cells that had been treated with shGFP or shPERK at 48 hpi.
(TIF)
