# Supplementary figures and images for: PKR-Like Endoplasmic Reticulum Kinase Is Necessary for Lipogenic Activation during HCMV Infection
Source: PLoS Pathog. 2013 Apr 4;9(4):e1003266. doi: 10.1371/journal.ppat.1003266 (PMC3617203; doi:10.1371/journal.ppat.1003266)

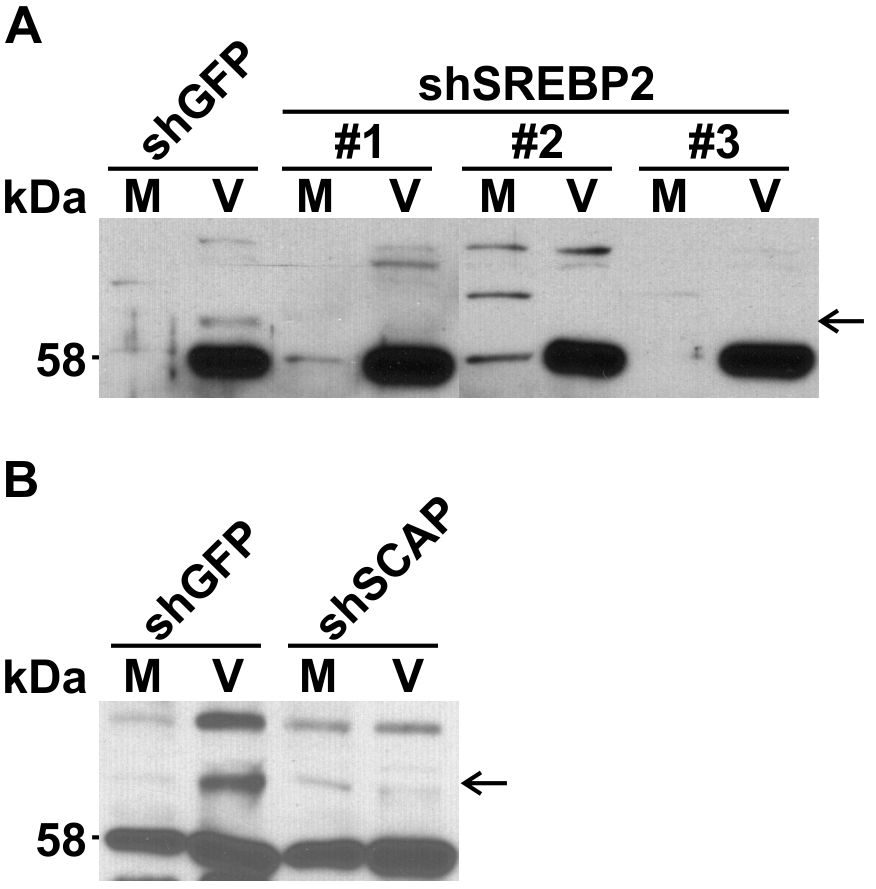

Supplement: Figure S1 — (A) Whole cell extracts were prepared at 48 hpi from HF cells treated with shGFP or three independent shRNAs targeting SREBP2 (TRCN0000020665, TRCN0000020667, TRCN0000020668) for three days and serum-starved for one day prior to mock- or HCMV-infection. Western analysis was performed by anti-SREBP2 antibody to determine the levels of the mature form of SREBP2. (B) The mature form of SREBP2 in SCAP-depleted cells. HF cells were treated with shGFP or shSCAP (TRCN0000078063) and infected with HCMV as described in (A), and then whole cell extracts were prepared. Western analysis was performed by anti-SREBP2 antibody to determine the levels of the mature form of SREBP2. M, mock infection; V, HCMV infection; the arrow indicates the mature form of SREBP2. (TIF) [file ppat.1003266.s001.tif]

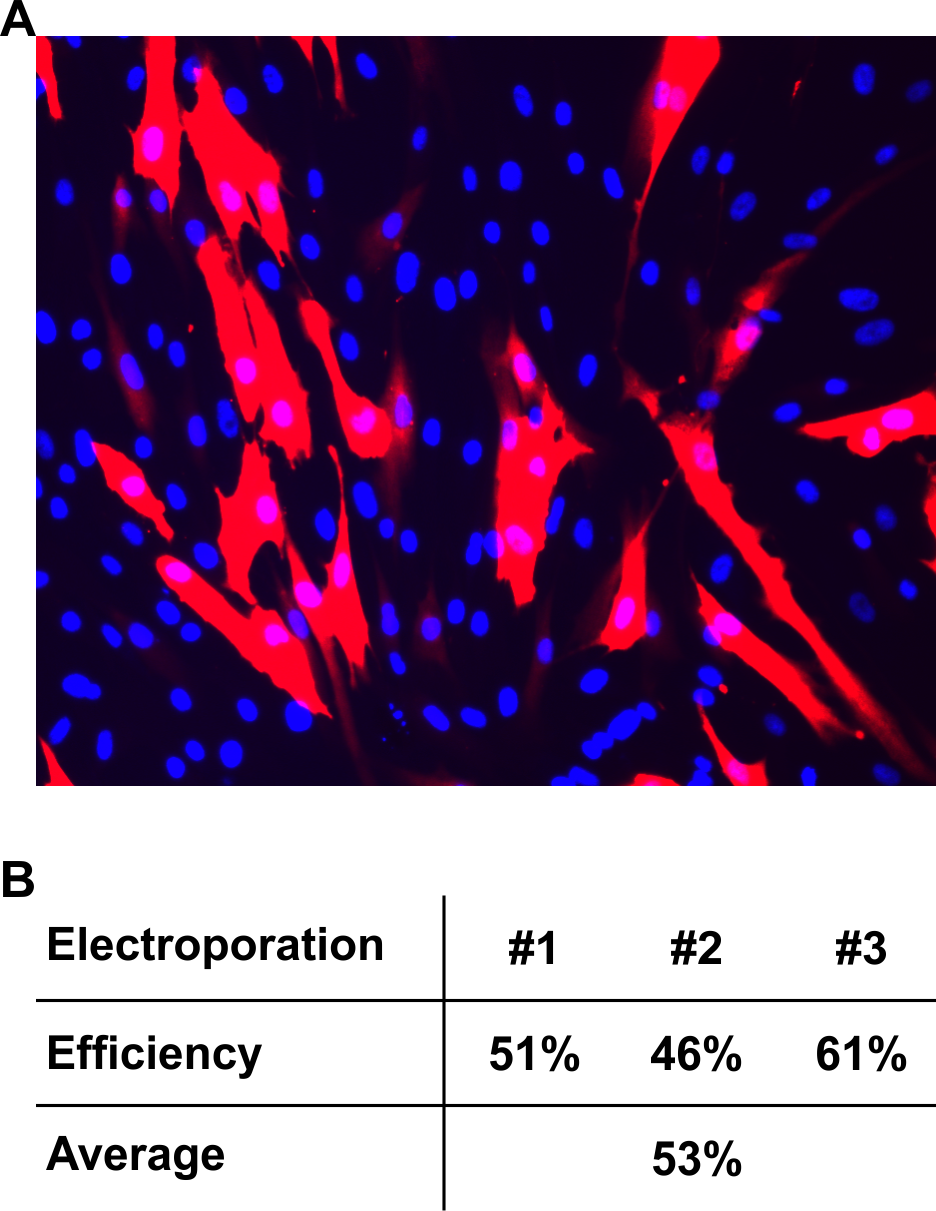

Supplement: Figure S2 — Transfection efficiency in HF cells via electroporation. (A) HF cells were transfected with a plasmid expressing RFP via electroporation. Two days after electroporation, cells on coverslips were fixed and stained with DAPI. The images were captured using a fluorescent microscope. (A) A representative image of electroporated HF cells. Red, RFP; blue, DAPI. (B) Average efficiency of three independent electroporations. After electropotation, fluorescent images were captured. Cells with RFP or DAPI signal were counted by Image-Pro 6.3 software and transfection efficiency was calculated. (TIF) [file ppat.1003266.s002.tif]

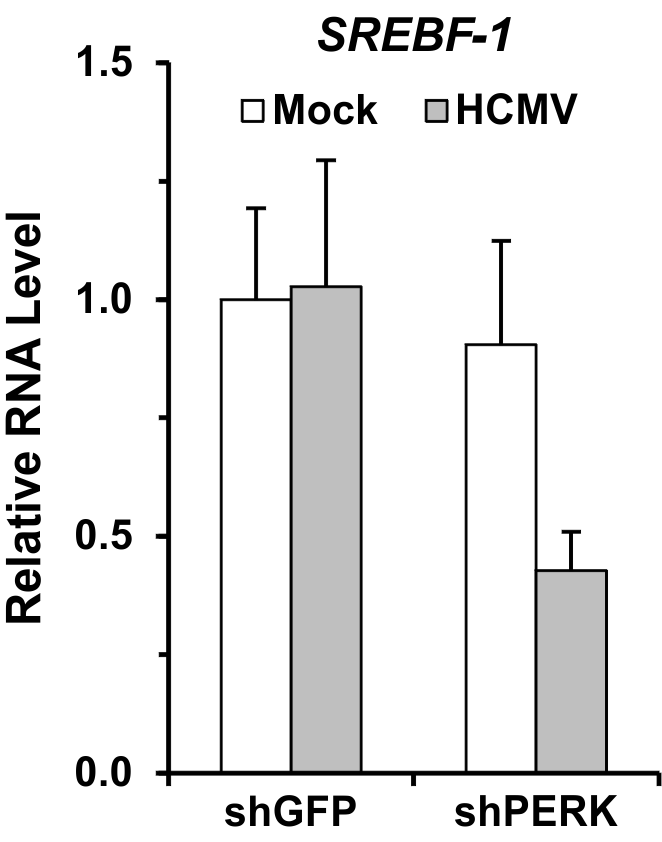

Supplement: Figure S3 — SREBP1 mRNA levels in PERK-depleted HF cells. mRNA levels of SREBP1 were determined by quantitative RT-PCR using total RNA extracted from mock- and HCMV-infected cells that had been treated with shGFP or shPERK at 48 hpi. (TIF) [file ppat.1003266.s003.tif]
